# The British Medical Association at Birmingham

**Published:** 1911-07-29

**Authors:** 


### THE BRITISH MEDICAL ASSOCIATION AT BIRMINGHAM.

The Annual Conference of the British Medical Association was opened at Birmingham on the 21st inst., when the Lord Mayor of the city (Alderman Bowater) welcomed the members who had assembled at the Midland Institute. "A whimsical intervention of a private member, as the Lord Mayor entered the Institute," reports the "Birmingham Daily Post," provided Alderman Bowater with a humorous introduction. "I should like to call your attention to the fact that a large quantity of gas is coming into the hall," observed the member, and the Lord Mayor bowed his acknowledgments. He hoped, he said, the member did not refer to what he was about to say, but rather to the gentlemen with whom he was better acquainted, and who would be speaking later. Mr. Bowater's speech referred to the advances in medical education which have been made in Birmingham and to the hospital arrangements which had been made in the city. Professor Saundby, the new President of the Association, pointed out that Birmingham could almost be said to have been the cradle of the Association. At the various sectional meetings many papers, none of them of outstanding importance, were read and discussed. Mr. Joseph Chamberlain has accepted the honorary membership of the Association.

Dr. Saundby delivered his presidential address on Tuesday, taking as his subject "The Present Position of the Medical Profession," in which he dealt mainly with the Insurance Bill club practice. Incidentally he gave his opinion on a State medical service. The nature of his comments on this point may be seen from such statements as "State doctors would be deficient in diligence and ability" and "State medical practitioners would be given the power to compel submission to treatment." Dealing with the hospitals he argued that the voluntary institutions should limit their work and not permit abuse, and pleaded for greater efforts to encourage scientific research.

### The Insurance Bill.

The main business of the representatives has been the discussion on the Insurance Bill. The statements made to the Press show that this discussion raised no new points. The following resolutions were passed: "That the Local Health Committees should be given any or all of the following powers—namely, to acquire, build, or equip their own sanatoriums or other buildings." "That the Council be instructed to use its best endeavours to have the £2 limit fixed in the Bill with provision for a lower limit to be fixed locally, but, failing that, to obtain, as best they can, the fixing of £2 as a maximum limit." "That an income limit should be fixed of £2 per week or such smaller sum as should be agreed, and that an insured person whose income exceeds such limit should not be entitled to obtain his medical benefit in the form of provision of medical attendance and treatment by the Local Health Committee in agreement with the Local Medical Committee, but should be required to take it in the form of a contribution to the cost of his attendance under such arrangements as he may make with his own doctor." The meeting strongly objected to Clause 57, and pressed for "provision for the compensation of those who will suffer loss in the value of their practices in the event of the Bill becoming law."

The Temperance Section held its usual meeting, and on Sunday several members addressed lay meetings in support of the crusade against alcohol. The arguments and facts adduced presented nothing that is novel or original, and the unfortunate overstatement of the case, which is so common at such meetings, was a marked feature of the addresses.

